# What Should Be Done Further to Protect the Public from the Increasing Empiricism Practiced in Our Large Cities

**Published:** 1883-07

**Authors:** C. M. Wright

**Affiliations:** Cincinnati


### What Should be Done Further to Protect the Public From the Increasing Empiricism Practiced in our Large Cities?

BY C. M. WRIGHT, D.D.S., CINCINNATI.

[Read before the Mad River Valley Dental Society.]

The philosophical use of the word empiricism has long been discarded by the medical profession; and the epithet "empiric," has become one of reproach. In the true sense, the empiric is one who learns by experience and observation. The burnt child has learned by empiricism to avoid the fire. In its philosophical meaning, it is a valuable aid to what may be distinguished as scientific knowledge.

What *we* mean to-day by empiricism can be definitely stated as the method of the dentist who advertises a "Company Dental Office," and offers to make a set of teeth for $10, and to extract a tooth *without pain* for fifty cents. Unfortunately, owing to the triple character of dentistry, we are brought into a certain competition with these men, and naturally wish to protect the public from them.

Dentistry, as we all know, is not a profession nor an art, nor a trade, but a curious mixture of the three. The list of subjects for discussion of any dental society in Europe or America proves this. This is why we are distinct in character, and cannot fully affiliate with physicians, or with sculptors, or with silversmiths and jewelers, and yet must be medical experts, artists, and mechanics in our own department of science and art. These men compete with us in our trade character, and you have appointed Prof. Taft and me to write essays on the subject, hoping, no doubt, for the suggestion of some legitimate way by which the public may be secured as our patron, and these men either driven to the wall, or forced to do as we desire. If these men simply advertise to make sets of teeth for $10, and do what they say they will do, and do not mislead the public, and conduct their business as shoemakers, clothiers, manufacturers and merchant princes do, although by so doing they violate the spirit and the letter of a code that *we* have adopted, I do not see that they are doing wrong, or that we have any *right* whatever, to interfere with them. If they injure our business, we must look out for ourselves, and adopt other and better means to seduce the public away from them. The great law of supply and demand is based on secure foundations in the science of political economy. If the people demand calico, the manufacturers cannot long force high-priced silk upon them. If the silk proves better, more durable, more beautiful, and *finally* cheaper than calico, the demand for it will increase, and calico will be worn only by the very poor. The manufacturer must *prove* the superiority of his silk, in order to create a demand on the part of the people. So must we. This is our work. The man who advertises calico teeth at $10, is not, on this account, a quack. When, however, he advertises "to extract teeth without pain," he is a quack and a charlatan. To offer inducements publicly (or privately) for foolish and ignorant people to come and be painlessly mutilated is quackery, and on a par with the crime of the medical abortionist. To painlessly extract a tooth, that dentistry should preserve; to painlessly excise a sore toe that surgery should save, is the height of injustice and wrong; and, to make a practice of this, or to advertise it, is quackery of the deepest dye. No true dentist would dare, in the presence of his own cultivated conscience, to extract a tooth that could be saved by the highest skill, whether he may possess that skill and science himself or not; any more than an oculist would dare to pluck out an eye, simply because he could do it painlessly, or, because the patient was too poor to pay for treatment, or too ignorant to properly value the organ, or too impatient to suffer treatment, or impertinent enough to threaten to "give the job" to another oculist. About the practice of advertising to extract teeth without pain, we, in the professional phase of our triple character, have a right to exert our most vigorous interference. Our regard for the health of the community demands it. Our regard for the dignity and nobility of dentistry demands it. Our regard for the future of the human face demands it. By the seductive nitrous-oxide gas, and our improved forceps—blessings of incalculable value in their proper use—an army of so-called dentists, are *steadily changing the anatomical proportions of the faces of the American people, besides crippling and preventing the important function of digestion, thereby making us a nation of dyspeptics, and entailing mental as well as physical deterioration.* I need not remind this learned society of the injurious effects of the loss of crowns, roots, and alveolar processes of the human jaws, and of the well marked natural consequences of this great destruction of tissue on the muscles of facial expression. The certain effects on the levator and depressor anguli oris, the depressor ala nasi, the orbicularis oris, the buccinator, the zygomatic and labial muscles are well known to sculptors and painters, if not to dentists. By the law of *evolution* what will the American face be in the future? In the light of art to-day, what sort of a figure would the typical mouth and lower half of an American face cut beside the model Greek face? And the American dentist, with his boastful mechanical and inventive genius, has not *culture* enough to blush at his own destructive work, because it is done deftly and painlessly. Then, we have invented nitro-glycerine, placed it in the hands of children, and sat and smiled at the use they make of it. Is this superior intelligence or imbecility? Here is where we should wake up to a desire "to protect the public from empiricism," whether practiced in large cities or remote villages. Let us stop *this* evil by legislation, or by any means we can. We have a right, aye! a *duty* to perform here, in sounding the alarm to the people—in publishing warnings to the public—in resorting to any means possible to us to take this dangerous bauble out of the hands of ignorant, and consequently, fearless quacks and charlatans. Let the Mad River Valley Dental Society begin the fight, and carry the war throughout the State and Nation. Let us begin the work of "protecting the public from the increasing empiricism" by devising a plan for the forcible removal of "laughing-gas" from the hands of unqualified men. Other work and legislation are simply selfishness and injustice, compared to this. We have a State Board of Dental Examiners. Would it not be well to compel *every dentist* who wishes to administer anæsthetics to apply to this board, and by a thorough examination, to satisfy it of his special qualifications on this particular point, and to receive a special certificate or "permit" from these officers of the State for the use of these agents? Make *this* a law and attach a penalty for the violation of it. This would be of special advantage to the people, and would be appreciated by them; for no matter how free and independent they may feel, they have no right to be injured by quacks, with the sanction of an honorable profession in its *professional character*. No dentist would feel himself injured, for, unless he is willing to qualify himself, he knows that he should not be allowed to risk the lives of his fellow men, and if he does not wish to administer anæsthetics, as many do not, his conscience would permit him to recommend the *qualified man*.

Appoint a committee at this meeting to confer with other committees from other societies, and push the subject into the legislature and before the people. In a short time we should feel that the man to whom we send our loved and valued patients, for the administration of nitrous-oxide gas, and the extraction of necessary teeth, was at least physiologist enough to know about systemic and pulmonary circulation, and medical man enough to be able to form a diagnosis of diseases, and expert enough to know what and how to apply restoratives for scientific reasons in cases of need. That we cannot be sure of this now, is a disgrace to us, and detracts largely from our boast that we are *professional gentlemen*, and that dentistry is a specialty of medicine, and a profession, instead of a trade. This should be my first suggestion. If it is not practiced, and I know that our government is not paternal in character, rather permitting Young America to play with any edge tool till scores of people have been killed, and then reluctantly taking the plaything away. I should suggest a combination of city dentists, of the better class, and the appointment by them of a thoroughly competent specialist for the administration, for them, of anæsthetics, and the extraction of teeth condemned to the forceps by themselves. These dentists should agree to support this appointed specialist, by giving him employment, at respectable and fixed fees, calling him to their offices when desirable, or sending, or accompaning patients to his rooms. He, on his part, should agree not to undertake any mechanical dental work, either in the operative or prosthetic departments, but to devote himself to his specialty, as a chloroformist does in a city hospital. In this way we alone would be responsible for the extraction of teeth, as we alone should be. In cities, where there are dental colleges, it seems to me that much might be done toward the suppression of this quackery, if a dental hospital, or infirmary, or poly-clinic, could be kept open the year round, for the administration of anæsthetics, the treatment of diseases of the teeth, etc., free to the poor. Operative and mechanical departments might also be kept open, with privilege of charging for operations just sufficient to pay expenses. This would be a boon to the poor, and advantage to the better class of dentists, and a training school for students, the value of which was so pointedly shown by our president, in his essay on Dental Colleges, at our last meeting.

I have suggested three plans. The first is somewhat similar to the method discussed by English dentists, and warmly advocated by many of the best men in London. You are familiar with the arguments of these distinguished men, and of such men, in our own country, as the late Dr. Barker, of Philadelphia, and Dr. Geo. Watt, of our society. The former in his paper on the "Abuse of Nitrous Oxide Gas," and the latter, in his many lectures and essays on the subject of "Painless Dentistry," Anæsthesia, "Nitrous Oxide Gas," etc., etc., have demonstrated the importance of a proper *qualification* of the man who may be trusted with these powerful agents.

The second and third plans are means that lie within our own hands entirely, and require simply harmony of action on our part. Feeling as I do, and as I have often expressed before, that the reckless extraction of the natural teeth and the substitution of *factory teeth*, is peculiar to our country, and a disgrace to us. I have offered these plans in pursuance of your invitation, hoping that something may be *done* to stem the current of physical *destruction*, and *false art*, that threatens to overwhelm us. I have approached the subject in reverence and modesty. If I have expressed myself dogmatically, I prithee charge it to my mental *diathesis*, and the love I bear ye.

